# Hans Georg Trüper (1936–2016) and His Contributions to Halophile Research

**DOI:** 10.3390/life6020019

**Published:** 2016-05-12

**Authors:** Aharon Oren

**Affiliations:** Department of Plant and Environmental Sciences, The Alexander Silberman Institute of Life Sciences, The Hebrew University of Jerusalem, Edmond J. Safra Campus, Jerusalem 91904, Israel; aharon.oren@mail.huji.ac.il; Tel.: +972-2-658-4951

**Keywords:** Hans Georg Trüper, halophilic, haloalkaliphilic, osmotic adaptation, compatible solutes, *Halorhodospira*, Archaea, *Natronomonas*

## Abstract

Prof. Hans Georg Trüper, one of the most important scientists in the field of halophile research, passed away on 9 March 2016 at the age of 79. I here present a brief obituary with special emphasis on Prof. Trüper’s contributions to our understanding of the halophilic prokaryotes and their adaptations to life in hypersaline environments. He has pioneered the study of the halophilic anoxygenic phototrophic sulfur bacteria of the *Ectothiorhodospira*—*Halorhodospira* group. Some of the species he and his group isolated from hypersaline and haloalkaline environments have become model organisms for the study of the mechanisms of haloadaptation: the functions of three major organic compounds – glycine betaine, ectoine, and trehalose – known to serve as “compatible solutes” in halophilic members of the Bacteria domain, were discovered during studies of these anoxygenic phototrophs. Prof. Trüper’s studies of hypersaline alkaline environments in Egypt also led to the isolation of the first known extremely halophilic archaeon (*Natronomonas* *pharaonis*). The guest editors dedicate this special volume of Life to the memory of Prof. Hans Georg Trüper.

## 1. Prof. Hans Georg Trüper—A Brief Curriculum Vitae

Prof. Dr. Dr. Dr. Hans Georg Trüper, who has made many important contributions to our understanding of the taxonomy, physiology, and ecology of halophilic microorganisms, passed away in Bonn, Germany, on 9 March 2016 [1,2]. His beloved wife Erika had died only a few weeks before.

Hans Trüper was born on 16 March 1936 in the village of Aumund near Bremen, Germany. He studied biology at the University of Marburg. Subsequently, he moved to the University of Göttingen where he studied the physiology and biochemistry of anoxygenic phototrophic bacteria under the guidance of Prof. Hans Günter Schlegel. This work resulted in a Ph.D. thesis on the CO_2_ fixation and the intermediary metabolism of *Chromatium okenii* [3]. He then spent periods of post-doctoral research with Prof. Holger Jannasch at the Woods Hole Oceanographic Institution, Massachusetts, and with Prof. Harry Peck at the University of Georgia, Athens, Georgia, USA. These studies on sulfur metabolism, and notably on the enzyme adenylylsulfate reductase, in photosynthetic sulfur bacteria were the topic of his “habilitation” thesis [4]).

In 1972, Hans Trüper was appointed professor of microbiology at the University of Bonn, where he established the Institute for Microbiology, today the Institute of Microbiology and Biotechnology. He retired in 2001.

Among the many important positions Prof. Trüper has held during his career, we can list the following:

1 He was a member of the Judicial Commission of the International Committee on Systematics of Bacteria-ICSB (now the International Committee on Systematics of Prokaryotes-ICSP) and Chairman of the Judicial Commission from 1990 to 1996.
2 He was a member of the editorial board of the International Journal of Systematic Bacteriology and its successor, the International Journal of Systematic and Evolutionary Microbiology, from 1990 to 2008, including a brief period in 2002 as Editor-In-Chief.
3 He was a founding member of the *Vereinigung für Allgemeine und Angewandte Mikrobiologie* (VAAM, the German Society for General and Applied Microbiology). Since 1986, he represented the VAAM within the Federation of European Microbiological Societies (FEMS) and the International Union of Microbiological Societies (IUMS).
4 He served terms as Vice President (1998–2001) and President (2001–2004) of FEMS.
5 He contributed to the 8th edition of Bergey’s Manual of Determinative Bacteriology and to the 1st and 2nd editions of Bergey’s Manual of Systematic Bacteriology.
6 He served on the editorial board and contributed articles to the first three editions of “The Prokaryotes”.

## 2. Prof. Hans Trüper’s Contributions to the Study of Halophilic Prokaryotes

**Figure 1:**
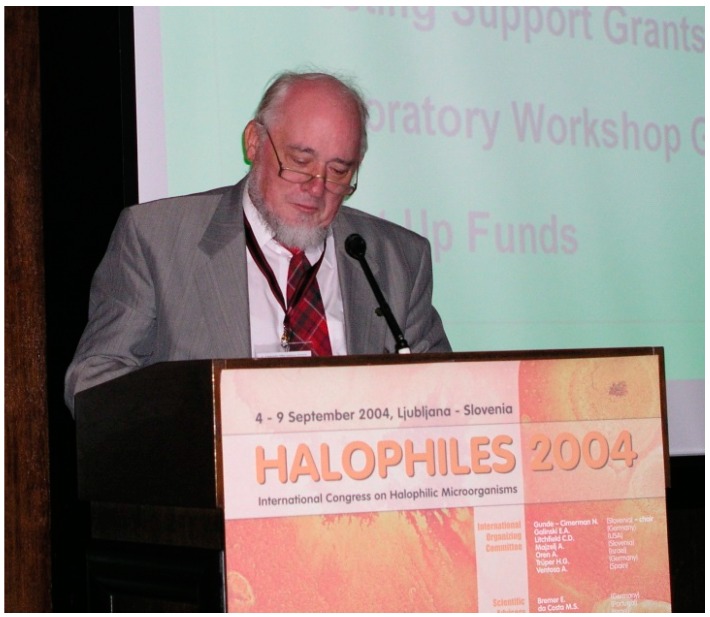
Prof. Hans Georg Trüper addressing the audience at the opening session of the Halophiles 2004 symposium in Ljubljana, Slovenia.

Hans Trüper has participated in the halophiles symposia held in Obermarchtal, Germany (1985), Alicante, Spain (1989), Sevilla, Spain (2001), and Ljubljana, Slovenia (2004) (Figure 1). He was a founding member of the ICSB/ICSP Subcommittee on the Taxonomy of Halobacteriaceae and served on that subcommittee for twenty years (1982–2002).

It may be assumed that his great interest in the halophiles and in hypersaline environments started during his post-doctoral studies with Holger Jannasch in Woods Hole, Massachusetts. Prof. Jannasch was one of the first microbiologists to study the alkaline hypersaline lakes of the Wadi Natrun in Egypt, and his study published in 1957 [5] stressed the importance of phototrophic sulfur bacteria and the microbial sulfur cycle in general in the functioning of that interesting ecosystem [6]. Later, Hans Trüper spent a period as a guest teacher at Ain Shams University, Cairo, which gave him the opportunity to visit the Wadi Natrun lakes, survey their microbiology with his students and coworkers [7,8], and isolate different haloalkaliphilic prokaryotes, phototrophs as well as heterotrophs, Bacteria as well as Archaea, from the site.

## 3. Isolation and Characterization of Novel Types of Halophilic Prokaryotes

The studies of samples from the Wadi Natrun lakes in Egypt led to the isolation and description of the following novel species of halophilic prokaryotes:

1 *Ectothiorhodospira vacuolata*, a moderately halophilic and alkaliphilic isolate, characterized by the presence of gas vesicles, and having bacteriochlorophyll *a* as its main photosynthetic pigment [9].
2 *Ectothiorhodospira* *abdelmalekii* [10], later renamed *Halorhodospira abdelmalekii* [11], an extreme halophile having bacteriochlorophyll *a* as its main photosynthetic pigment.
3 *Ectothiorhodospira halochloris* [12], later renamed *Halorhodospira halochloris* [11], an extremely halophilic isolate containing bacteriochlorophyll *b*. This species later became a model organism for the study of the modes of osmotic adaptation of halophilic phototrophic Bacteria (see below).
4 A heterotrophic Gram-positive bacterium strain WN13 [13], later described as *Bacillus* *haloalkaliphilus* [14] and then renamed *Alkalibacillus haloalkaliphilus* [15].
5 The extremely halophilic alkaliphilic aerobic archaeon *Natronobacterium pharaonis* [16], later renamed *Natronomonas pharaonis* [17], described as an organism with a very low magnesium requirement, a property in which the strain differs from most neutrophilic members of the class Halobacteria.

## 4. Studies on the Mechanisms of Osmotic Adaptation in Halophilic Prokaryotes

The newly isolated halophilic/haloalkaliphilic strains from the Wadi Natrun lakes proved excellent model organisms for the study of the mechanisms of osmotic adaptation of members of the domain Bacteria to life at high salt concentrations by Hans Trüper and his students. In the late 1970s–early 1980s, it was known that the halophilic eukaryotic alga *Dunaliella* uses glycerol as “compatible solute” and thus avoids the accumulation of high concentrations of toxic ions within its cytoplasm. The “salt-in” strategy of Archaea of the class Halobacteria was recognized already in the early 1970s, but nothing was known about the strategies of osmotic adaptation in the moderately or extremely halophilic anoxygenic phototrophs and other highly salt-tolerant and salt-requiring members of the Bacteria domain.

The discovery of glycine betaine as the main osmotic solute in *Halorhodospira halochloris* [18] provided the first evidence of the importance of this simple compound as an osmotic solute in many microorganisms, even in those where *de novo* biosynthesis of the compound is not possible. *H. halochloris* can not only synthesize glycine betaine, but it can also take up the compound from the medium [19]. Another extremely important osmotic solute first discovered in *H.* *halochloris* was identified as 1,4,5,6-tetrahydro-2-methyl-4-pyrimidinecarboxylic acid, a cyclic amino acid now known as ectoine [20]. Its biosynthetic pathway starting with l-aspartate 4-semialdehyde was eludicated quickly after the discovery of the compound [21], and a sensitive analytical method for its detection was devised [22]. A third novel organic osmotic solute was found in *H. halochloris*: trehalose [23]. Upon dilution stress, excess trehalose is degraded intracellularly by a trehalase [24], while excess amounts of glycine betaine and ectoine are excreted from the cells. Loss of glycine betaine may even exceed the quantities required by the dilution stress, and the compound can subsequently be recovered by active transport through the cell membrane [25].

At the time, the role of organic compatible solutes in the osmotic adaptation of heterotrophic Bacteria was not yet fully realized. Results obtained with the haloalkaliphilic strain WN13, now known as *Alkalibacillus haloalkaliphilus*, made it clear that it cannot contain high intracellular ionic concentrations, as 0.5–1 M NaCl strongly inhibits the activity of key enzymes such as isocitrate dehydrogenase and malate dehydrogenase [13]. Now we know that the species synthetizes ectoine as its osmotic solute and can also accumulate glycine betaine from its medium [14].

The early studies on organic “compatible” solutes by Hans Trüper and his students quickly showed that such compounds may find biotechnological applications. Survival of *Escherichia coli* during drying and storage was considerably improved in the presence of compatible solutes [26]. These studies have led to the wealth of information known today about the beneficial action of such solutes and their biotechnological exploitation for diverse applications.

These and other studies on the role of compatible organic solvents in osmotic adaptation in prokaryotes have been summarized by Hans Trüper in a number of review papers [27,28].

## 5. Further Biochemical and Taxonomic Studies of Halophilic Prokaryotes

Halophilic prokaryotes and hypersaline environments also proved interesting study material to learn more about the sulfur cycle and the enzymes involved in sulfur transformation processes. Cytochrome c-551 was shown to be involved in the oxidation of elemental sulfur in *H. abdelmalekii* [29]. Prof. Trüper’s attempts to isolate dissimilatory sulfate-reducing bacteria from brine and core muds of Atlantis II Deep and Discovery Deep, hot brines on the bottom of the Red Sea, did not succeed, but positive enrichment cultures were obtained from the transition zone of Atlantis II brines and seawater. One of the strains isolated could grow up to 170 g/L salt [30]. This strain was unfortunately not preserved.

All new isolates of halophilic anoxygenic phototrophs from the Wadi Natrun lakes were subjected to taxonomic studies, comparing their properties with *Ectothiorhodospira* and *Halorhodospira* species recovered from other environments. Detailed studies have targeted the lipids [31] and the carotenoid pigments [32].

Hans Trüper also contributed to a study of the potential of different halophilic Archaea of the class Halobacteria to grow anaerobically using dimethylsulfoxide or trimethylamine-*N*-oxide as the electron acceptors in respiration [33]. 

All these studies provided an excellent basis for review papers on the ecology and ecophysiology of different types of halophilic and halotolerant microorganisms adapted to life in hypersaline environments of different kinds [34,35,36].

At the halophiles conference in Alicante in 1989, Hans Trüper made an interesting effort to detect coherent trends in the strategies used for osmotic adaptation by different groups of halophilic and halotolerant microorganisms based on their taxonomic and phylogenetic affiliations [37]. At the time, our understanding of the tremendous diversity in haloadaptation mechanisms within the microbial world was still limited, so apparent correlations could still be observed. However, with the advancing knowledge and with the in-depth study of more model organisms, it became ever more difficult to formulate general trends to describe the relations between halophily, taxonomy, and phylogeny. The state of the art was re-evaluated by the author of this essay at the halophiles symposia in Colchester in 2007 and in Storrs in 2013, and the picture became less and less coherent [38,39]. Even within a single genus of extreme halophiles such as the genus *Halorhodospira* (Gammaproteobacteria), so extensively studied by Hans Trüper in the early years of compatible solute research, disparate strategies were reported. While *H. halophila* maintains high intracellular KCl when grown at the highest salt concentrations and has an acidic proteome, *H. halochloris* does not accumulate KCl and appears to rely entirely on organic solutes to provide the necessary osmotic balance; its proteins do not show a great excess of acidic over basic amino acids [40]. Thus, it may well be impossible now to obtain a coherent picture with clear correlations between phylogenetic affiliation and modes of salt adaptation as Prof. Trüper tried to achieve.

## 6. Hans Georg Trüper and Prokaryote Nomenclature

In view of his contributions to halophile science, two new species of halophilic prokaryotes have been named to honor Hans Trüper: *Halobacillus trueperi* and *Natranaerobius* *trueperi*. The first is an endospore-forming member of the *Firmicutes*, isolated from the Great Salt Lake, Utah [41]; the second is a true polyextremophile isolated from the Wadi Natrun lakes in Egypt, and it combines halophilic, alkaliphilic, and thermophilic properties [42].

The topic of biological nomenclature was very dear to Prof. Trüper. He had a great passion for the classical languages (and for other languages as well), and he was always eager to share his knowledge to help others less well versed in linguistics to find suitable names for new taxa of microorganisms. This led to the publication in 1999 of a review paper with practical guidelines how to name new genera and species of prokaryotes [43] and to the preparation of a new version of Appendix 9—Orthography to the International Code of Nomenclature of Prokaryotes [44]. In addition, after his retirement, he assisted many colleagues who needed suitable names for new organisms they wished to describe.

As thanks for all the help, a number of other, non-halophilic prokaryotes have been named in his honor:

1 One family: the Trueperaceae with the genus *Truepera* as type genus [45].
2 Three genera: *Truepera*, *Trueperella*, and *Hanstruepera*. *Truepera* is a genus of radiation-resistant bacteria affiliated with the Deinococcus/Thermus phylum; it currently has a single species: *T. radiovictrix* [45]. The genus *Trueperella*, currently with five species, was separated from the genus *Corynebacterium* (Actinobacteria) [46]. *Hanstruepera*, with the single species *H. neustonica*, is a zeaxanthin-producing member of the family Flavobacteriaceae isolated from estuarine water [47].
3 Three species: *Rhodospira* *trueperi*, *Thiobaca* *trueperi*, and *Sphingomonas trueperi*. The first two are non-halophilic anoxygenic phototrophs, belonging respectively to the Alphaproteobacteria and the Gammaproteobacteria classes [48,49]. The last one is a heterotrophic alphaproteobacterium [50].

## 7. Hans Georg Trüper—The Historian

From his early years as a high school student, Hans Trüper had had a keen interest in history. He managed to perform academic history studies in parallel with his occupation as professor of microbiology and his many other academic duties in Bonn. His studies of the regional history of the Elbe-Weser area in the Middle Ages resulted in a second Ph.D. degree from *Hochschule Vechta* (now *Universität Vechta*) under the guidance of Prof. Bernd Ulrich Hucker (1998).

The community of halophile scientists seized the opportunity to become acquainted with Prof. Trüper’s historical insights at the Halophiles 2001 symposium in Sevilla, where he presented the closing lecture entitled “*Cum grano salis*—Salt in the history and life of mankind. An overview with emphasis on Europe.” The text of this presentation was published in the symposium proceedings [51]. This paper also contains a fascinating section on “Salt in the etymology of prokaryote names,” in which Hans Trüper provides many ideas, some of which have not or have little been used yet, for those who wish to include Latin or Greek roots related to salt in new names of prokaryote genera and species.

## 8. Awards and Honors Received by Hans Georg Trüper

During his long career, Prof. Trüper has received a large number of awards and honors. Here is a list of the most important ones:

1 He was elected as a corresponding member of the Academy of Sciences in Göttingen (1987).
2 He was made an honorary member of the *Sociedad Española de Microbiología*—the Spanish Society for Microbiology (1997).
3 He received the Bergey’s Medal for long-term excellent merits in bacterial taxonomy (1999).
4 He received an Honorary Doctorate from the Faculty of Biology and Chemistry, University of Bremen (2002).
5 He became an Honorary Fellow of the Hebrew University of Jerusalem (2003).
6 He was elected as member of the Wittheit (Academy of Sciences) in Bremen (2003).
7 The *Vereinigung fu¨r Allgemeine und Angewandte Mikrobiologie* (VAAM) made him an honorary member (2008).
8 He was elected a Life Member of the International Committee on Systematics of Prokaryotes at its meeting in Istanbul (2008) [52].

In view of all his contributions to halophile science and other aspects of microbiology, the editors of this special issue of “Life” dedicate this proceeding volume of the Halophiles 2016 symposium to the memory of Prof. Hans Georg Trüper.
